# An epigenetic human cytomegalovirus infection score predicts viremia risk in seropositive lung transplant recipients

**DOI:** 10.1080/15592294.2024.2408843

**Published:** 2024-10-03

**Authors:** Fei-Man Hsu, Rashmi P. Mohanty, Liudmilla Rubbi, Michael Thompson, Harry Pickering, Elaine F. Reed, John R. Greenland, Joanna M. Schaenman, Matteo Pellegrini

**Affiliations:** a Department of Molecular, Cell and Developmental Biology, University of California Los Angeles, Los Angeles, CA, USA; b Institute for Quantitative and Computational Biosciences – The Collaboratory, University of California Los Angeles, Los Angeles, CA, USA; c Department of Medicine, University of California San Francisco, San Francisco, CA, USA; d Department of Pathology and Laboratory Medicine, David Geffen School of Medicine, University of California Los Angeles, Los Angeles, CA, USA; e Department of Medicine, David Geffen School of Medicine, University of California Los Angeles, Los Angeles, CA, USA

**Keywords:** Cytomegalovirus, DNA methylation, epigenetics, kidney transplantation, lung transplantation, biomarker

## Abstract

Cytomegalovirus (CMV) infection and reactivation in solid organ transplant (SOT) recipients increases the risk of viremia, graft failure and death. Clinical studies of CMV serostatus indicate that donor positive recipient negative (D^+^/R^−^) patients have greater viremia risk than D^−^/R^−^. The majority of patients are R^+^ having intermediate serologic risk. To characterize the long-term impact of CMV infection and assess viremia risk, we sought to measure the effects of CMV on the recipient immune epigenome. Specifically, we profiled DNA methylation in 156 individuals before lung or kidney transplant. We found that the methylome of CMV positive SOT recipients is hyper-methylated at *loci* associated with neural development and Polycomb group (PcG) protein binding, and hypo-methylated at regions critical for the maturation of lymphocytes. In addition, we developed a machine learning-based model to predict the recipient CMV serostatus after correcting for cell type composition and ancestry. This CMV episcore measured at baseline in R^+^ individual stratifies viremia risk accurately in the lung transplant cohort, and along with serostatus the CMV episcore could be a potential biomarker for identifying R^+^ patients at high viremia risk.

## Introduction

Human cytomegalovirus (CMV) is a β-herpesvirus that typically resides in the host in a latent form without causing overt symptoms [1]. CMV infects around 60–90% of adults worldwide [2], and the global CMV seroprevalence rate ranges from 45 to 100% among women of reproductive age [1]. In certain conditions, such as a weakened immune system, CMV can reactivate into the lytic phase and cause viremia, leading to symptomatic infection [2].

The characteristic owl’s eye inclusions caused by CMV infection were first seen in stillbirths in 1910 and later among patients undergoing solid organ-transplantation (SOT) [3]. Seropositive organ donors (D^+^) have approximately a 78% chance to transmit CMV to seronegative recipients (R^−^) [4]. About 40% of seropositive organ recipients (R^+^) reactivate latent CMV during immunosuppression therapy post-transplantation, and those with seropositive donors can also be reinfected with new strains of CMV [4,5]. CMV reactivation can cause viremia, allograft rejection, and end-organ diseases post-transplantation [4,6,7]. Serologic risk groups are determined based on the CMV serostatus of recipient and donor combinations. A study of 653 renal transplant patients indicated that D^+^/R^−^ (high sero-risk) has a CMV viremia odds ratio 87.46 compared to the D^−^/R^−^ (low sero-risk), and R^+^ (intermediate sero-risk) slightly increases the risk but does not arrive at statistical significance [8]. Given the fact that more than half of the human population are CMV seropositive with intermediate sero-risk, a comprehensive CMV viremia risk assessment method would be advantageous to most of the SOT patients.

Peripheral blood DNA methylation is one of the most studied epigenetic modifications and reflects the cumulative record of lifetime exposures that are associated with several non-commutable diseases, including Alzheimer’s, multiple sclerosis, type 2 diabetes, systemic lupus erythematosus and cardiovascular disease. In many cases methylation signatures have high predictive value and could be used to predict health outcomes [9,10]. The epigenetic clock [11–13] and epigenetic pacemaker [14] have been widely studied and predict chronological ages. However, less is known about the association of DNA methylation with infections. Recently, the Milieu Intérieur Consortium reported that latent CMV infection drives DNA methylation variation in blood through the regulation of host transcription factors in a cell-composition-mediated manner [15]. This raises the possibility that end-organ diseases post-transplantation and other sequelae of SOT are also affected by alterations in the DNA methylome of immune cells.

To investigate the DNA methylation perturbation due to CMV infection, we leveraged longitudinal pre- and post- transplant biorepository samples from research participants undergoing kidney and lung transplant at UCLA and UCSF and applied targeted-bisulfite sequencing (TBS-seq) to profile their peripheral blood mononuclear cells (PBMCs). In contrast to previous epigenome-wide association studies (EWAS) that used the Infinium methylation array, our approach allows us to select regions of interest that are more likely to be impacted by CMV infection. We built a model from the pre-transplant (PreTx) samples that can predict CMV serostatus even post-transplantation (PostTx). We identified CMV-associated CpG sites after correcting for the effect of cell composition and ancestry. Finally, we demonstrated that our epigenetic CMV measure named CMV episcore serves as an epigenetic biomarker to estimate time to viremia following SOT.

## Materials and methods

### Human subjects

#### Kidney transplantation cohort

The study procedures, informed consent, and data collection documents were reviewed and approved by the Institutional Review Board of the UCLA (IRB#11-001,387). Chart review was performed to acquire demographic and clinical data including results of CMV PCR surveillance. Participants provided blood samples on the day of transplantation (PreTx) and 3 months after (PostTx).

#### Lung transplantation cohort

Lung transplant candidates were recruited to participate in a longitudinal database and biorepository, as previously described [16]. The study procedures, informed consent, and data collection documents were reviewed and approved by the Institutional Review Board of the UCSF (IRB#13-10,738). Participants provided blood samples prior to induction immunosuppression during the time of transplantation (PreTx) and at a clinically indicated bronchoscopy around 12 months after (PostTx).

## Blood samples

8 ml of blood was drawn into the ACD tube. After Ficol density gradient centrifugation, PBMCs were separated isolated and cryopreserved in FCS/DMSO.

### Targeted bisulfite sequencing (TBS-seq)

#### Probe design

The probe panel design is based on the criteria to include CpG *loci* that 1) covers DNA methylation clock age estimators [12,13], 2) has cell-type specificity, and 3) locates in the promoter regions (−1000 to + 250bp from TSS) of viral response genes [17]. Biotinylated probes covering the selected CpG *loci* were synthesized by IDT (Integrated DNA Technologies). The coordinates of the targeted regions (GRCh38) are listed in Supplementary Table S1.

#### Library preparation

Genomic DNA was extracted from PBMCs using phenol-chloroform method [18]. 500ng genomic DNA was sheared and subject to end-repair, A-tailing and ligated with methylated adaptors. Purified libraries were hybridized to biotinylated probes and subjected for bisulfite conversion (Zymo Cat# D5030). Captured DNA was PCR amplified with KAPA HiFi HotStart Uracil^+^ (Cat# KK2801) into a final TBS-seq library. Library quality was evaluated using TapeStation with the high-sensitivity D1000 tape (Agilent Cat#5067–5584). A comprehensive TBS-seq protocol is demonstrated in [17].

#### TBS-seq data processing

Adapter sequences were trimmed off from the raw reads using Cutadapt [19] and only reads with minimum 30bp were kept for downstream analysis. Reads were aligned to GRCh38 reference genome using bsbolt align function and the duplicated reads were marked with *samtools markdup* function before calling methylation using bsbolt callmethylation function [20]. CGmaps from all samples were aggregated into one methylation count matrix using *bsbolt aggregatematrix* function with parameters *-min-coverage 10 -min-sample 1.0*.

## Cell type deconvolution

A reference-based cell type deconvolution approach was used to estimate cell type composition with DNA methylation profiles [21]. To recapitulate cell type composition of PBMC, WGBS dataset from 6 cell types: B cell, CD4 T cell, CD8 T cell, NK cell, naïve T cell and monocyte were acquired from GSE186458 [22], and neutrophil band cell’s methylation profiles were from the BluePrint database [23]. In total, 34 WGBS profiles from each cell type with replicates were included (Supplementary Table 2). Cell type-specific differentially methylated regions (DMRs) were identified by one-vs-all comparisons using metilene [24] with the criteria to find DMRs that are1) at least 500bp, 2) with the delta methylation level < −30%, and 3) with a false discovery rate (FDR) < 0.05. Cell type-specific CpG sites were further subtracted from each TBS-seq sample with bedtools intersect function and used as input files to deconvolute. A non-negative least square approach was applied to every TBS-seq profile and regress to the WGBS references for coefficient estimation. The detailed deconvolution data could be found in Supplementary Figure S1.

### Methylation modeling

#### Multivariate multiple linear regression (MMLR)

Per recruit i, the methylation status of targeted locus j is denoted as $M_{\mathrm{ij}}$. Suppose every $M_{\mathrm{ij}}$ is described by k methylation-associated traits, *i.e*. multivariate, of each recruit $T_{\mathrm{ik}}$, that are weighted by per site coefficient $C_{\mathrm{kj}}$, the methylation model is formulated as Equation 1

(1) $$M_{\mathrm{ij}}=T_{\mathrm{ik}}\times C_{\mathrm{kj}}\left\{\begin{array}{c} i\in \mathrm{number}\;\mathrm{of}\;\mathrm{recruits}\\ j\in \mathrm{number}\;\mathrm{of}\;\mathrm{CpGloci}\\ \;\;\;\;\;\;\;k\in \mathrm{number}\;\mathrm{of}\;\mathrm{traits} \end{array}\right.$$

This model represents a system of equations in which $T_{\mathrm{ik}}$ and $M_{\mathrm{ij}}$ are known variables. Our goal is to derive critical CpG sites for each trait, *i.e*. the unknown $C_{\mathrm{kj}}\;$that represents characteristics of sites, and could be achieved by solving Equation 2 as,

(2) $$C_{\mathrm{kj}}=T_{ik}^{†}\times M_{\mathrm{ij}}$$

Here, $T_{ik}^{†}$ is derived through Moore-Penrose pseudoinverse of $T_{\mathrm{ik}}$.

#### Leave-one-out cross validation (LOOCV)

To avoid over-fitting, for each biological sample, a separate MMLR model was trained with the rest samples to derive C, and the trait prediction is made as,

(3) $$T_{\mathrm{ik}}=M_{\mathrm{ij}}\times C_{kj}^{†}$$

Here, $C_{kj}^{†}$ is the Moore-Penrose pseudoinverse of $C_{\mathrm{kj}}$.

#### Identification of CMV serostatus-associated CpG sites

To identify statistically significant associations between CMV serostatus and the methylation per site, for each locus we estimated the significance of the coefficients described below,

(4) $$\;\;\text{\textbackslash{}1}y_{m}=\beta_{0}+\beta_{1}x_{m1}+\beta_{2}x_{m2}+\ldots +\beta_{n}x_{\mathrm{mn}}\;+\varepsilon$$

Here, $y_{m}$ is the methylation level at locus m, $x_{n}$ are the explanatory variables including age, sex, CMV serostatus, cohort, cell type PCs, and ancestry PCs. $\beta_{0}$ is the y-intercept, $\beta_{n}$ is the coefficient for each explanatory variable, and ε is the error. For each CpG site $y_{m}$, p values from the model per explanatory variable $x_{n}$ were derived and adjusted for multiple hypothesis testing with Benjamini-Hochberg correction. CpG sites with adjusted p value <0.05 regarding CMV serostatus as x were determined as CMV associated sites.

#### Cox proportional hazards (coxph) model

The sero-risk per subject was categorized into high (D^+^/R^−^), intermediate (R^+^) and low (D^−^/R^−^). Both sero-risk and the CMV episcore derived from the MMLR prediction of CMV status were treated as covariates of the Coxph model to estimate the rate of CMV viremia over the tracking time, i.e. 3 months for the kidney cohort and 12 months for the lung cohort. The Coxph regression analysis was performed with R package ‘survival’ and plotted with R package ‘ggsurvplot.’

## Functional enrichment analysis

Site-level GO enrichment analysis was performed using GREAT [25] with CMV associated sites’ coordinates as foreground and all TBS-seq captured CpG sites as background. Gene-level GO enrichment analysis was conducted with Enrichr [26]. TFBS enrichment analysis were performed using Cistrome [27].

### RNA-seq

#### Library preparation

RNA was extracted from PBMC. mRNA libraries were constructed using KAPA RNA HyperPrep Kit following the manufacturer's instruction (Cat#KK8540). RNA was sheared and primed with oligos for cDNA synthesis. After adapter ligation and PCR amplified, the final library was quantified and accessed quality with TapeStation.

#### RNA-seq data processing

Reads were aligned to reference genome GRCh38 using STAR using default parameters [28]. A gene count table was generated using featurecount [29]. Gene count matrix was first normalized and DEGs were identified using R package DEseq2 (Love *et al*, 2014). Genes with adjusted p value <0.05 and at least 2-fold difference between CMV serostatus were considered differentially expressed.

## Results

### Latent CMV infection alters the host blood methylome

**Figure 1.**
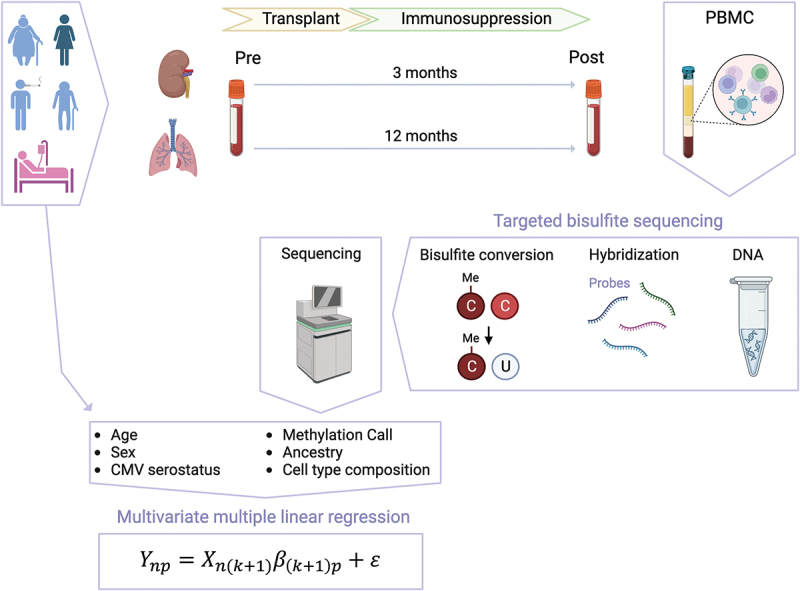
Schematic of this CMV study.

The study design is illustrated in Figure 1. Participants in the kidney cohort underwent transplantation between April 2015 and September 2021, and the lung cohort was recruited between September 2015 and November 2020. Blood samples were obtained on the day of transplantation from 84 lung and 72 kidney participants, and a subset of 57 participants also provided blood samples after SOT at about 12 and 3 months. The participant characteristics are shown in Table 1 and the correlation between traits is shown in Supplementary Figure S2a.

Renal transplant candidates at UCLA were recruited to participate before kidney transplantation, while lung transplant candidates listed for transplantation were recruited at UCSF. Participants provided blood on the day of transplantation and followed up after 3 months (kidney) and 12 months (lung) for PostTx sampling. PBMCs were isolated and genomic DNA was extracted for TBS-seq. After sequencing, the DNA methylation profiles were analyzed and modeled with the multivariate multiple linear regression (MMLR) model. The illustration is created with BioRender.

**Table 1.** Participant characteristics.

	Kidney	Lung
N	72	84
Age median [IQR]	52.3 [40.5, 59.0]	63.8 [58.4, 67.3]
Sex = Male, N (%)	43 (59.7)	34 (40.5)
Ethnicity, N (%)		
Asian	7 (9.9)	4 (4.8)
Black	10 (14.1)	6 (7.1)
Hispanic	38 (53.5)	21 (25.0)
Other	2 (2.8)	2 (2.4)
White	14 (19.7)	51 (60.7)
Double transplant, N (%)	59 (83.1)	80 (95.2)
Cytomegalovirus serostatus, N (%)		
D^−^/R^−^	6 (8.3)	12 (14.3)
D^−^/R^+^	18 (25.0)	13 (15.5)
D^+^/R^−^	17 (23.6)	19 (22.6)
D^+^/R^+^	31 (43.1)	33 (39.3)
CMV viremia after Tx, N (%)	18 (25.0)	27 (32.1)
PostTx sequenced, N (%)	18 (25.0)	2 (2.4)
Post-transplant/Pre-transplant, N (%)	50/72 (69.4)	7/84 (8.3)

To profile DNA methylation we used targeted bisulfite sequencing (TBS-seq). We designed the TBS-seq probe panel to capture sites that are associated with age [12,13] along with cell type specific regions and the promoters of genes that mediate responses to viral infections (Supplementary Table S1). Our assay captured 37,379 CpG sites with a minimum coverage of 10X across all samples (Supplementary Figure S2b).

**Figure 2.**
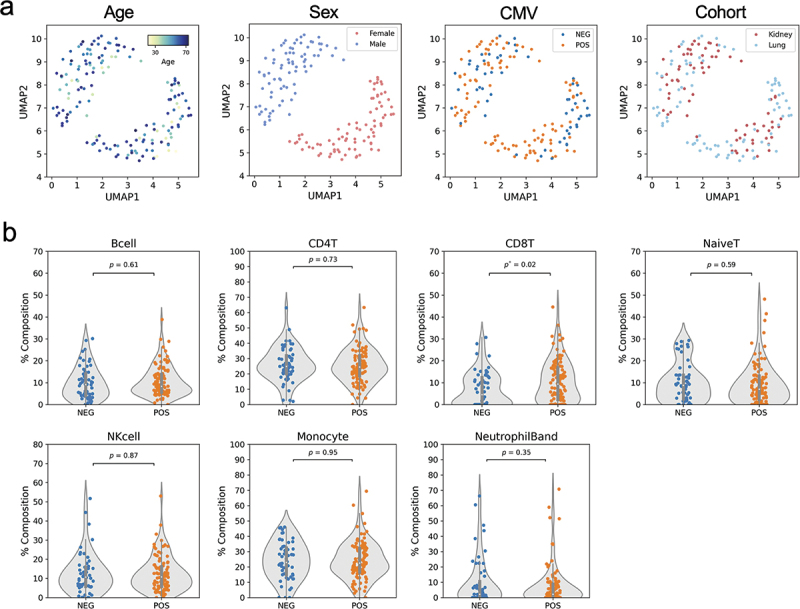
CMV latent infection alters the blood methylome and cell-type composition.

To identify the factors that drive variation in methylation across our cohort we used Uniform Manifold Approximation and Projection (UMAP), to visualize our samples in two dimensions. In PreTx samples, we found that in addition to sex, which is the main factor driving clustering of samples, CMV serostatus further separates individuals (Figure 2a). Principal component analysis (PCA) of DNA methylation also shows correlation with CMV serostatus to PC1 (Supplementary Figure S2c). These results suggest that latent CMV infection has a significant impact on the host methylome.

(a) UMAPs show the naïve clustering of DNA methylation groups with sex as the major and CMV serostatus as minor factor differentiating samples. (b) Cell-type composition estimated from DNA methylation suggests CD8 T cells increased in CMV seropositive subjects.

### CD8 T cell composition is associated with CMV serostatus

It is well established that a significant driver of changes in DNA methylation in PBMCs is cell type composition. To account for this effect, we carried out cell type deconvolution using cell-type specific DNA methylation sites (see Materials and Methods and Supplementary Figure S1) to estimate the percentage of B cells, CD4, CD8 and naïve T cells, along with NK cells, monocytes, and neutrophils in each sample. Among these cell types, only CD8 T cells were significantly increased in CMV^+^ patients (Figure 2b).

### DNA methylation serves as a predictor variable of CMV serostatus

We next asked if DNA methylation could be used to predict CMV serostatus. We trained a Leave One Out Cross Validated (LOOCV) penalized logistic regression model in a cohort of 156 PreTx samples (Supplementary Figure S3a). The trained DNA methylation model excluding the test individual was first cross-validated to optimize the hyper parameter and then correctly predicted recipient CMV status for 115 participants (74.7%) (p value <0.001***, Spearman’s rank correlation), corresponding to an area under curve (AUC) of 0.68.

**Figure 3.**
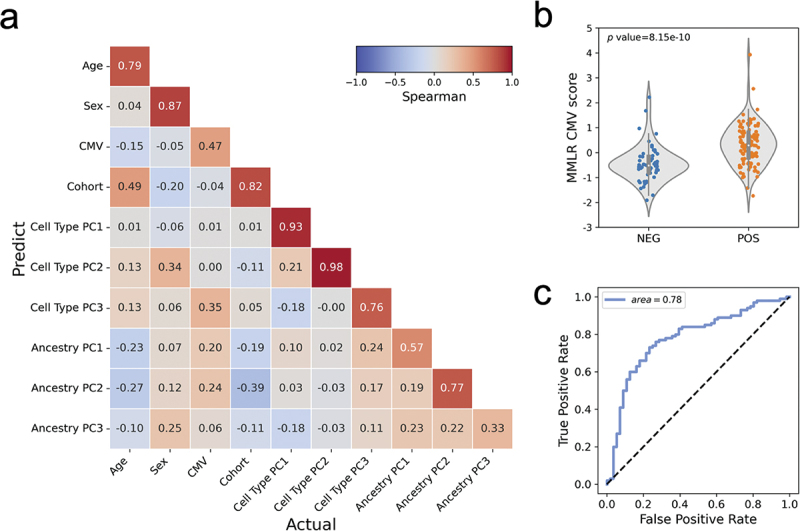
Multivariate multiple linear regression methylation model.

To determine whether the effects of CMV are mediated by changes in cell type composition and other covariates we used LOOCV to assess a multivariate multiple linear regression model (MMLR) with DNA methylation as the response variable and multiple scaled traits including age, sex and CMV serostatus as dependent variables (Figure 3). We also included into the MMLR model our estimated cell type PCs (Supplementary Figure S3b) and ancestry PCs derived from genotypes inferred from the targeted bisulfite sequence data. By inverting the model, we could also predict the values of each trait for each individual from their DNA methylation profiles (see Materials and Methods).

(a) Spearman’s correlation matrix of the actual (x-axis) and predicted (y-axis) traits. (b) Violin plot with data points shows the predicted value, CMV episcore, has a significant difference in distribution between CMV seropositive and seronegative recipients. (c) ROC curve of the MMLR prediction model for CMV serostatus.

Figure 3a shows the spearman correlation between the predicted and actual values of traits in our MMLR model. We see that the predicted levels of CMV serostatus have a 0.47 correlation coefficient with the actual value (p value < 0.001***, Figure 3b). We named the predicted CMV serostatus from the MMLR model the ‘CMV episcore’ and find that the associated AUC is 0.78 (Figure 3c). These results indicate that DNA methylation can be used to predict CMV serostatus even when other covariates are considered.

We next asked if CMV serostatus of individuals could be predicted after SOT. We used the PreTx LOOCV MMLR model to predict the CMV serostatus of the 56 PostTx individuals, and the predict-actual correlation coefficient is 0.46 with AUC 0.81 and p value < 0.01** (Supplementary Figure S3c). To avoid overfitting in this analysis, we left out the PreTx individual when predicting their paired PostTx sample. This result suggests that CMV episcores are stable across 3–12 months after organ transplantation.

### Characterization of CpG sites that are associated with CMV latent infection

**Figure 4.**
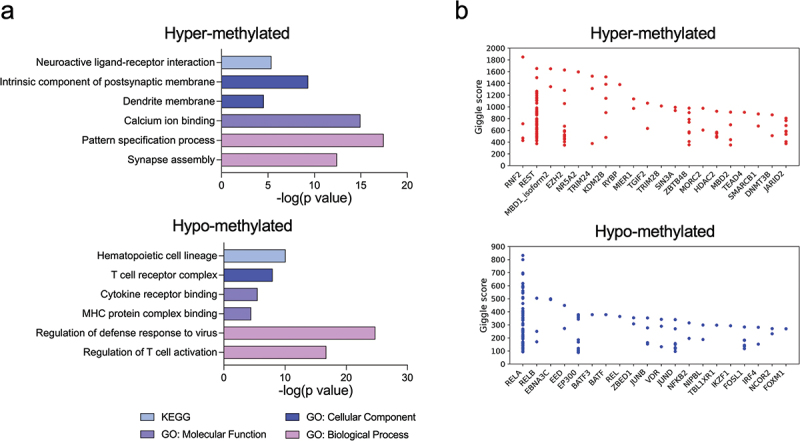
CMV latent infection is associated with hypermethylation of neural system genes and hypomethylation of immune response genes.

We next examined CpG sites associated with the CMV serostatus. We modeled the methylation level per site using a multiple linear regression model and characterized CpG *loci* with adjusted p value <0.05 (see Materials and Methods). The sign (+ or -) of the coefficient of the CMV serostatus determines whether the sites are hyper- and hypo-methylated in CMV seropositive recipients. We found 2,217 CpGs that are hyper-methylated in CMV seropositive recipients and 1,535 CpGs that are hypo-methylated (Supplementary Figure S4a). Genes that are proximal to these sites are listed in Supplementary Table 3 (hyper-methylated) and Supplementary Table S4 (hypo-methylated). Hyper-methylation of genes in promoters typically reduces gene expression. The hyper-methylated CpGs are enriched in neural functions such as synapse assembly and neuroactive ligand-receptor interaction (Figure 4a). Gene ontology analysis of hypo-methylated genes in CMV seropositive patients, on the other hand, showed enrichment in hematopoietic cell lineage, T cell receptor complex, major histocompatibility complex (MHC) protein binding, and T cell activation. Interestingly, we also saw both hyper- and hypo-methylation of the ACE2 locus, the receptor for the spike glycoprotein of the human coronavirus SARS-CoV-2 (severe acute respiratory syndrome coronavirus 2), as shown in Supplementary Figure S4b.

(a) Gene ontology of hyper-methylated (top) and hypo-methylated (bottom) CpG sites. (b) Enrichment of TFBSs of hyper-methylated (top) and hypo-methylated (bottom) CpG sites.

To further characterize these differentially methylated sites we asked if they were enriched for the binding of specific transcription factors that might be regulating their methylation levels. This analysis was performed using Cistrome [27] (Figure 4b). CMV latent infection associated hyper-methylated CpG sites are enriched in PcG proteins including RNF2, EZH2, KDM2B and JARID2. By contrast, CpG sites that are hypo-methylated in CMV seropositive cases mainly reside in the transcription factor binding sites (TFBSs) of 1) the NFkB pathway, including RELA and RELB, and 2) AP-1 transcription factor members including BATF, BATF3, JUNB, JUND and FOSL1.

### Gene expression related to T cell receptor signaling and activation is up-regulated in CMV seropositive patients

**Figure 5.**
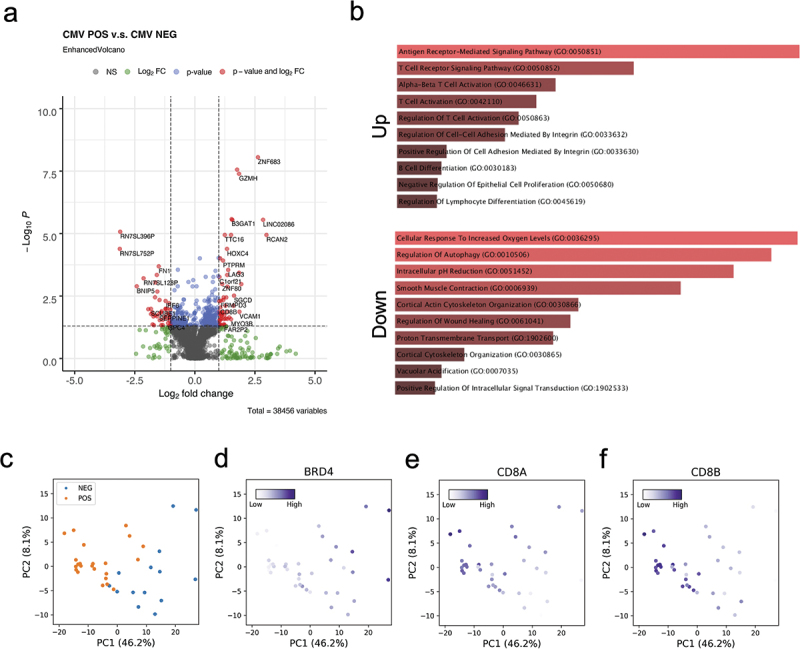
Transcriptomic impact of CMV latent infection.

To determine whether the DNA methylome alterations we observed to be associated with CMV seropositivity are accompanied by gene expression changes, we performed bulk RNAseq in a subset of 23 CMV seropositive and 14 CMV seronegative individuals in the kidney transplant cohort. 194 up- and 121 down-regulated genes were identified between CMV positive and negative individuals (Figure 5a, Supplementary Tables S5&6). The CMV seropositive up-regulated genes are enriched in the antigen receptor mediated signaling pathway, T cell receptor signaling pathways, and T cell activation (Figure 5b), and these GO terms are similar to those we found enriched among the hypo-methylated genes (Figure 5b). The down-regulated genes are enriched in cellular response to increased oxygen level, regulation of autophagy, and intracellular pH reduction (Figure 5b).

(a) Volcano plot of differentially expressed genes regarding CMV serostatus. (b) Gene ontology of up- and down-regulated genes in CMV seropositive subjects. (c) PCA of transcriptomes shows separation according to CMV serostatus. (d-f) Feature plots of BRD4, CD8A and CD8B that are both differentially methylated and expressed.

As shown in the PCA, CMV seronegative status is separated from CMV seropositivity by the PC1 axis, suggesting the latent CMV infection also impacts the human blood transcriptomes (Figure 5c). We identified 5 genes that are both differentially methylated and expressed based on CMV serostatus. One of these, BRD4 (Bromodomain-containing protein 4), is a histone acetylation reader that regulates CMV latency and reactivation [30], and whose methylation is significantly increased in CMV seropositive patients [15]. We found 1 CpG site downstream of BRD4 to be hyper-methylated, and BRD4 is down-regulated in CMV seropositive patients (Figure 5d). Interestingly, we found CD8A and CD8B, subunits of CD8 antigen receptor complex on T cell surfaces, are hypo-methylated and up-regulated in CMV seropositive participants (Figures 5e,f).

### CMV epigenetic score as a biomarker to predict CMV viremia

**Figure 6.**
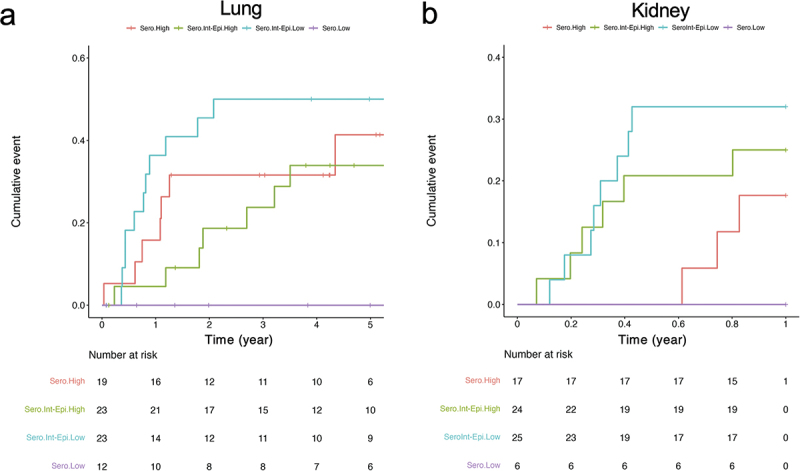
Time-to-viremia event analysis stratified by CMV serostatus risk group and CMV episcore.

Traditional serologic risk assessment trichotomized SOT patients into high (D^+^/R^−^), intermediate (R^+^) and low (D^−^/R^−^) based on the serostatus in donor and recipient to avoid CMV viremia and its sequelae such as graft rejection and death. We therefore asked if the CMV score estimated from the MMLR model (CMV episcore) could be used to improve risk assessment for CMV viremia. Specifically, we hypothesized that increasing CMV-specific immune responses, as captured by this methylation score, would be associated with decreased risk of viremia. In both transplantation cohorts, we measured the hazard ratio of CMV viremia over 12 months (kidney) and 5 years (lung). The Cox proportional hazard regression models show that the CMV episcore (HR 0.74, 95% CI 0.57–0.96, p* = 0.02) is a significant covariate in the lung cohort. Graphically, we showed that when the R^+^ group is stratified by CMV episcore, the CMV episcore low group had worse survival than the high episcore group (Figure 6a). In the kidney model, the CMV episcore was not statistically associated with time-to-viremia (Figure 6b), but still from the cumulative survival plot the CMV episcore low group bears the worst hazard ratio. The differences in statistic power between cohorts could be due to the duration of the follow-up window and the immunosuppression therapies (see Discussion). In conclusion, the CMV episcore is a potential biomarker to predict CMV viremia and is especially useful for the R^+^ patients.

(a) In the lung cohort (n = 77, 46 events), CMV episcore was associated with increased CMV viremia rates (HR 0.74, 95% CI 0.57–0.96, p* = 0.02). (b) Time to CMV viremia is shown for the renal transplant recipient cohort (n = 72, 18 events) shows no statistical difference with CMV episcore as a covariate but still it further frames the sero-risk intermediate group into 2 layers.

## Discussion

We identified a significant impact of CMV latent infection on the methylome of PBMC in two centers and across individuals before receiving lung or kidney transplant. Differential methylation patterns went beyond what could be explained by inferred cell compositional differences, affecting genes involved in neural programming and T cell differentiation. We also identified CMV associated changes in RNA expression. Interestingly, a DNA methylation-based CMV episcore was able to predict CMV viremia risk in intermediate sero-risk (R^+^) lung transplant recipients, and the hazard ratio of patients with low CMV episcores even surpass the high sero-risk D^+^/R^−^ group. In kidney transplant recipients with intermediate sero-risk (R^+^), lower CMV episcores also result in shorter time to viremia, while not showing statistical significance. Since the CMV episcore derived PreTx is associated with CMV viremia risk after SOT, a patient with a low CMV episcore has a high CMV viremia risk independently of the matching donor’s CMV serostatus and the resulting CMV prophylaxis should be calibrated accordingly.

We found that CMV infection was linked to increased abundance of CD8 T cells. These observations are consistent with prior studies using flow cytometry that showed the number of CD8 TEMRA is increased in CMV seropositive patients [31–34]. Since the impact of CMV infection on the epigenome could be mediated by multiple factors, we constructed a MMLR model of the methylome that accounted for factors, including age, sex, cell type composition and ancestry. This model allows us to predict CMV serostatus when controlling for these covariates. The MMLR model predicted CMV value, named CMV episcore, is associated with the actual CMV serostatus (*R* = 0.47, Figure 3a). This intermediate correlation holds for dichotomized samples (Figure 3b) and results in a classification power with an AUC of 0.78 (Figure 3c), suggesting the epigenetic perturbation by latent CMV infection is a distributed effect but not localizes to a small fraction of CpGs.

The functional enrichment analyses of CMV associated CpG sites point to hyper-methylation in neural systems genes and hypo-methylation in the immune system genes (Figure 4a). Genes that are hyper-methylated in CMV seropositive individuals include LTBP3, a TGF-β component reported in another study [15], whose mutation/loss-of-function results in hearing loss and otosclerosis [35]. KDM2B is also hyper-methylated and is related to congenital ocular defects [36]. This is consistent with the observations that CMV is one of the leading causes of congenital vision defect and hearing loss in newborns from infected mothers, but it is still unclear if these neural defects are related to DNA methylation at these *loci*.

Hyper-methylated CMV associated CpG sites are also enriched in TFBSs of PcG proteins (Figure 4b). EZH2 is a lysine methyltransferase that methylates H3K9 and H3K27 and represses gene transcription. Together with its regulators KDM2B and JARID2, this PcG complex has been reported to repress GFI1, the MIEP transcriptional repressor of CMV [37]. Hyper-methylated in PcG targeted transcription factor binding sites might release GFI1, repress MIEP, and maintain CMV latent infection from entering the lytic phase. We also found TRIM28 binding sites are hyper-methylated in CMV seropositive patients (Figure 4b). A previous study showed that CMV could establish latency in hematopoietic stem cells (HSCs) resulting in lifelong infection [38], and that TRIM28, also known as KAP1, is responsible for switching off viral genes in stem cells to maintain latency [39]. These data are consistent with the hypothesis that TRIM28 regulates CMV latency through DNA methylation.

Genes that are hypo-methylated in CMV seropositive individuals include the T cell receptor subunits CD3D and CD3G, and the co-receptors of CD8 T cells, CD8A and CD8B (Figure 4a). Hypo-methylated CMV associated CpG sites are also enriched in TFBSs of NFkB and AP-1 (Figure 4b). NFkB and AP-1 are both proinflammatory TFs triggered by cytokines and act downstream of T cell receptors (TCRs) to activate T cells [40–43]. The fact that these sites are hypo-methylated in CMV positive individuals, suggests that the corresponding immune responses might be up-regulated. Hypo-methylation at these *loci* was also associated with concordant up-regulation of related genes/pathways (Figure 5). These results indicate that CMV latent infection may maintain the hosts’ immune system primed, especially the cellular immunity, through DNA methylation.

CMV has been reported to be highly prevalent in HIV (Human immunodeficiency virus)-infected subjects, and the coinfection is associated with the risk of cardiovascular and cerebrovascular events [44]. Recent cohort studies showed CMV seropositivity is a risk factor of severity and hospitalization due to SARS-CoV-2 [45,46]. In our study we found that ACE2, the SARS-CoV-2 receptor, is hypo-methylated in CMV seropositive individuals. Although ACE2’s expression is low and not increased in the PBMC samples we examined, we hypothesize that the CMV/SARS-CoV-2 superinfection in cell lines reported previously [47] could potentially be regulated by DNA methylation.

We also found that the CMV episcore could improve stratifications of CMV viremia risk in lung transplant recipients who are R^+^ with intermediate serologic risk. Increased CMV episcore was associated with decreased viremia risk, suggesting that this score broadly assesses CMV immunity and may be a biomarker of viremia risk in this population. In the kidney cohort, a low CMV episcore in the intermediate sero-risk (R^+^) group was nominally the highest risk, but this difference did not achieve statistical significance. In fact, the R^+^ group has a higher viremia risk than the high sero-risk group (D^+^/R^−^), and this could be because R^+^ patients receive 0 to 3 months CMV prophylaxes whereas the sero-risk high patients receive 6 months of CMV prophylaxis in kidney transplant recipients. We note that all lung transplant recipients were targeted for lifelong CMV prophylaxes. In addition, there were fewer CMV events and shorter follow up time in the kidney cohort, which diminished its statistical power. In conclusion from both cohorts, CMV episcore helps access the CMV viremia risk in hosts facing second infections and could represent the DNA methylation-based trained immunity [48].

## Conclusions

Our study reveals the long-term impact of CMV latent infection on the host methylome. Besides describing the differential methylation in CMV seropositive and seronegative patients, we also quantify the PreTx DNA methylome as a CMV episcore to access the viremia risk after SOT and could be beneficial to the broad CMV seropositive organ transplant recipients. As described by the Waddington landscape of cell fate decision [49], DNA methylation may play ‘permissive’ roles in CMV reactivation, congenital disease development, and host immune system priming.

## Limitations of the study

The aim of this study is to decipher the impact of CMV infection on the host DNA methylome. Our study has a few limitations. First, the clinical samples were drawn at different timepoints, and the sequencing libraries were constructed and sequenced separately. We therefore made ‘cohort’ a covariate in the MMLR model to resolve this issue. Second, the patients received lung or kidney transplant and therefore already had chronic diseases which might affect DNA methylation. Another limitation is that the time to viremia estimation using the CMV episcore is only significant in the lung cohort. Whether it is applicable to other SOT studies remains to be addressed with more cohorts. Lastly, although this measure could be beneficial to most of SOT patients with intermediate sero-risk (R^+^), applying TBS-seq to every patient might not be feasible. A more specific assay targeting fewer *loci* identified in this study and easier to apply needs to be developed in the future.

## Supplementary Material

Supplemental Material

SupplTable1.xlsx

SupplTable2.xlsx

SupplFigure3.tiff

SupplTable6.xlsx

SupplTable5.xlsx

SupplFigure1.tiff

SupplTable3.xlsx

SupplFigure2.tiff

SupplTable4.xlsx

SupplFigure4.tiff

## Data Availability

Sequencing data produced in this study are available in Gene Expression Omnibus upon publication. The kidney cohort is under the accession number GSE250536 (https://www.ncbi.nlm.nih.gov/geo/query/acc.cgi?&acc=GSE250536), and the lung cohort is under the accession number GSE253562 (https://www.ncbi.nlm.nih.gov/geo/query/acc.cgi?acc=GSE253562). The code for MMLR model is deposited to GitLab (https://gitlab.com/fmhsu0114/cmvepiscore).
