# Supplementary material for: An epigenetic human cytomegalovirus infection score predicts viremia risk in seropositive lung transplant recipients
Source: Epigenetics. 2024 Oct 3;19(1):2408843. doi: 10.1080/15592294.2024.2408843 (PMC11451273; doi:10.1080/15592294.2024.2408843)
Supplement: Supplemental Material [file KEPI_A_2408843_SM5228.docx]

# Supplementary Information

Figure S1. Supporting data of cell type deconvolution.

Figure S2 Demographic traits correlation and DNA methylation records from TBS-seq.

Figure S3. Supporting methylation models.

Figure S4. CMV associated CpG loci.

Table S1. TBS-seq probe coordinates.

Table S2. Reference WGBS dataset for cell type deconvolution.

Table S3. Hyper-methylated genes.

Table S4. Hypo-methylated genes.

Table S5. Up-regulated genes.

Table S6. Down-regulated genes.
