# Supplementary figures and images for: An epigenetic human cytomegalovirus infection score predicts viremia risk in seropositive lung transplant recipients
Source: Epigenetics. 2024 Oct 3;19(1):2408843. doi: 10.1080/15592294.2024.2408843 (PMC11451273; doi:10.1080/15592294.2024.2408843)

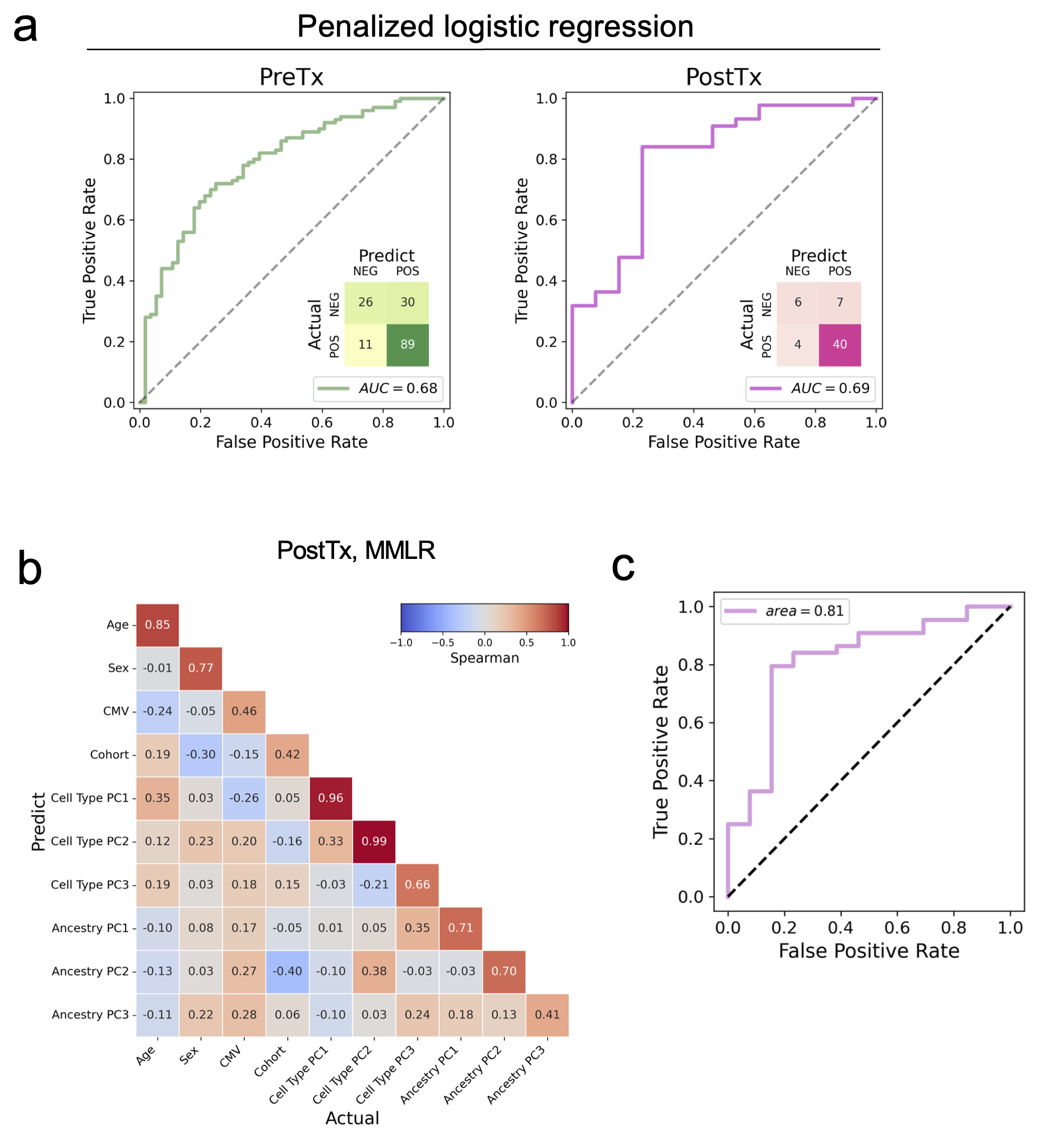

Supplement: SupplFigure3.tiff [file KEPI_A_2408843_SM4933.tiff]

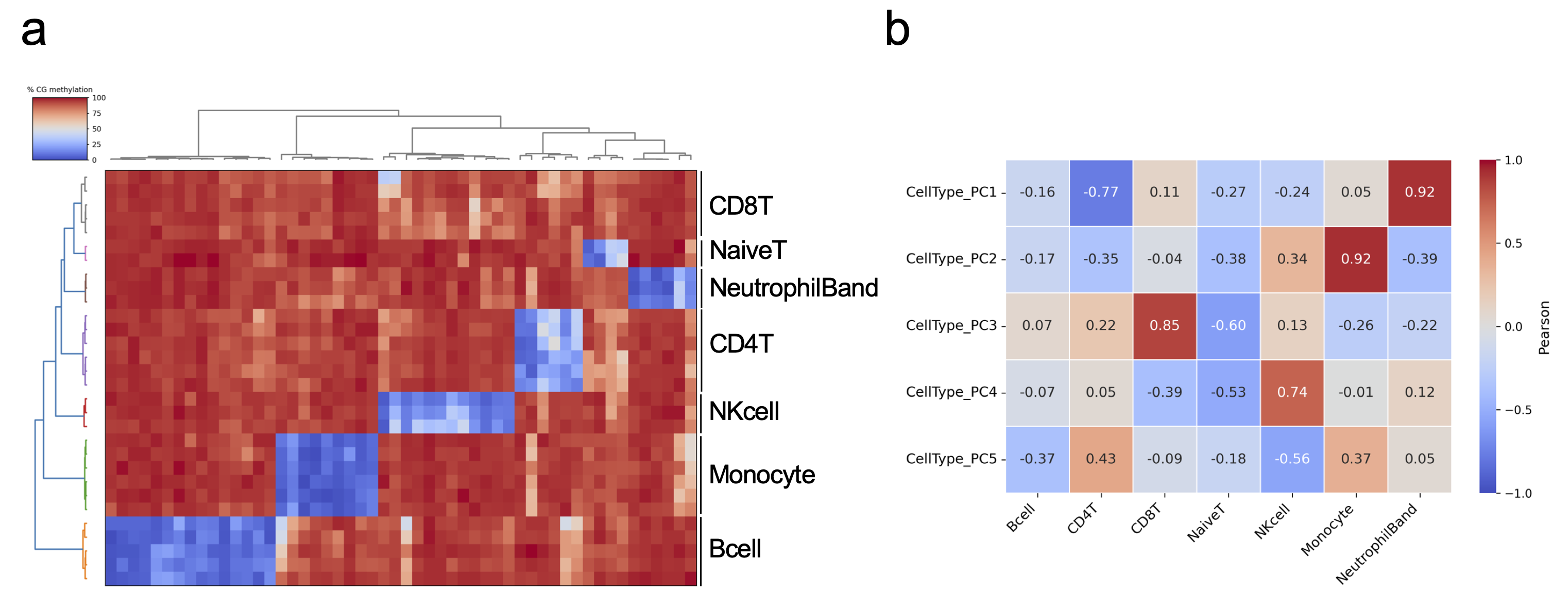

Supplement: SupplFigure1.tiff [file KEPI_A_2408843_SM4930.tiff]

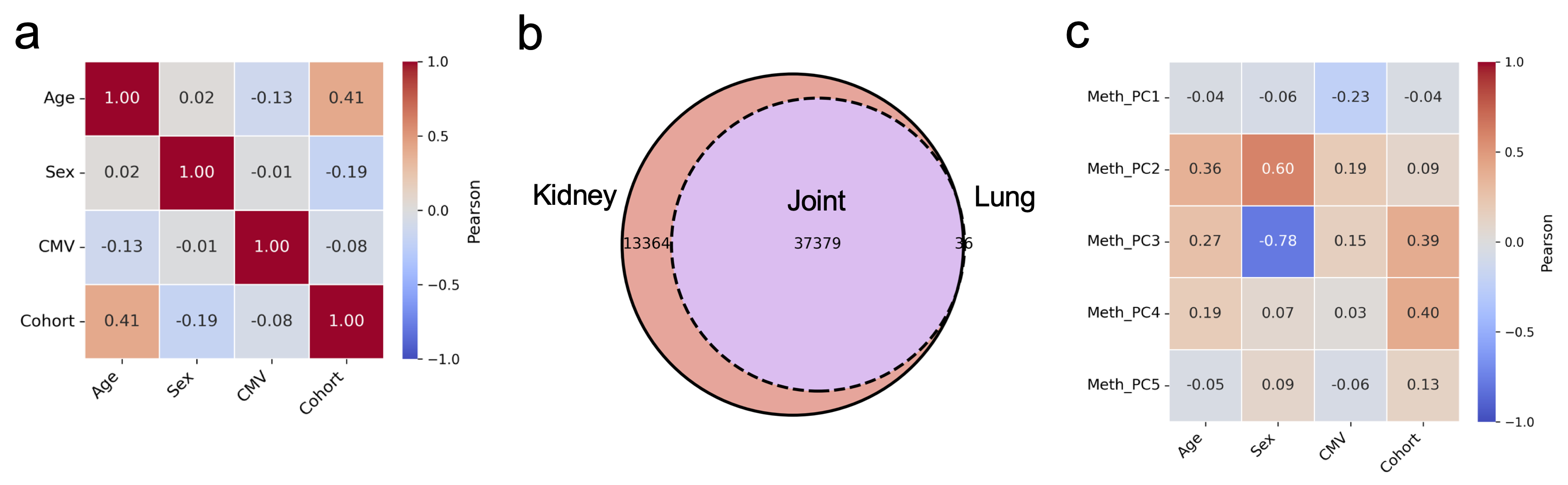

Supplement: SupplFigure2.tiff [file KEPI_A_2408843_SM4928.tiff]

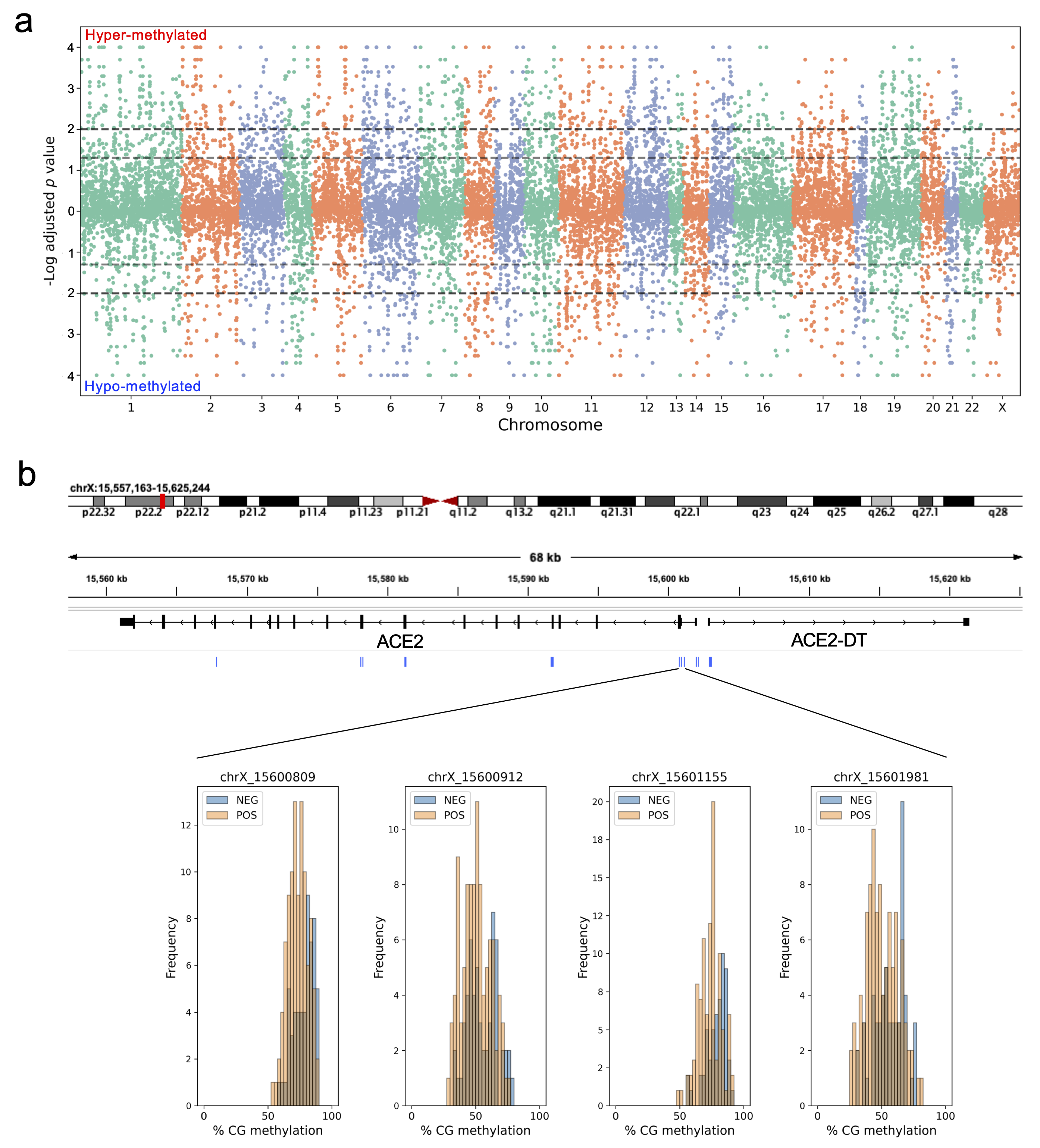

Supplement: SupplFigure4.tiff [file KEPI_A_2408843_SM4926.tiff]
